# Predicting COVID-19 booster intentions among college students using the health belief model: advancing health promotion strategies for uptake

**DOI:** 10.3389/fpubh.2024.1395941

**Published:** 2024-10-17

**Authors:** Hannah Priest Catalano, Julianne Christofora, Keith Richards, Katherine Hyatt Hawkins Shaw, Kevin Kiser

**Affiliations:** ^1^School of Health & Applied Human Sciences, University of North Carolina Wilmington, Wilmington, NC, United States; ^2^School of Communication, East Carolina University, Greenville, NC, United States; ^3^George Mason University, Fairfax, VA, United States; ^4^Department of Biology & Marine Biology, University of North Carolina Wilmington, Wilmington, NC, United States

**Keywords:** COVID-19, vaccination, health belief model, behavioral intention, college students

## Abstract

**Background:**

COVID-19 remains a significant public health threat. The primary purpose of this study was to test the health belief model (HBM) constructs in predicting COVID-19 booster intentions of college students.

**Methods:**

A total of 285 students enrolled at large public university in the Southeastern U.S., who were 18 years and older, completed an online survey to assess COVID-19 vaccination status, prior or current COVID-19 infection, demographics, and HBM constructs.

**Results:**

Over three quarters of the sample (81.4%, *n* = 232) was fully vaccinated, 2.1% (*n* = 6) was partially vaccinated, and 16.5% (*n* = 47) was unvaccinated. Furthermore, 53.4% (*n* = 124) of students who self-reported being fully vaccinated also reported receiving the booster vaccine. Nearly half of the sample (49.1%, *n* = 140) self-reported previously or currently testing positive for COVID-19. Results of the stepwise multiple regression indicated the HBM constructs of perceived benefits (*β* =0.596; *p* < 0.001) and cues to action (*β* =0.275; *p* < 0.001) were significant predictors of respondents’ behavioral intention to receive the COVID-19 booster in the next 6 months. The significant predictors at step 2 accounted for 64.6% [*R*^2^ = 0.646, *F* (2, 111 = 101.331, *p* < 0.001)] of the variance in behavioral intention to get the COVID-19 booster in the next 6 months.

**Conclusion:**

Practitioners developing HBM-based interventions to enhance COVID-19 booster intentions among college students should tailor health promotion strategies that target perceived benefits and cues to action. Although some of the HBM constructs were not statistically significant in the prediction model, they should not be entirely discounted in health promotion practice. Instead, practitioners should focus on supplemental strategies to improve those domains in college students.

## Introduction

In May 2023, the U.S. government declared that the public health emergency associated with COVID-19 had come to an end (1). Although no longer considered a public health emergency, the risks associated with COVID-19 remain real and the opportunity to learn from this pandemic and what influences the actions of those at risk remains an important area of study. The CDC (2) recommends everyone over 6 months of age and older getting and staying up to date with the COVID-19 vaccine. Other prevention strategies, such as wearing a mask in public areas where COVID-19 hospital admission levels are medium or high, limiting person-to-person interactions, washing hands as often as possible, avoid touching eyes, nose, and mouth, covering coughs and sneezes, disinfecting touched surfaces, improving ventilation and filtration, and moving indoor activities outdoors, are still viable precautions (3). The COVID-19 vaccination is highly effective at preventing people from becoming seriously ill, being hospitalized, and dying from the infection (4). In fact, the COVID-19 vaccines are the safest and most reliable means of protecting oneself from the complications of COVID-19 (4).

The CDC (5) bases their vaccine recommendations on several factors including the individual’s age, how long it has been since their last dose, and in some cases, they base their recommendation on which COVID-19 vaccine an individual initially received. The vaccines elicit an immune response which safeguards individuals without subjecting them to the morbidity and mortality risks associated with COVID-19 or the potential long-term complications following infection (4). To receive the most protection, the CDC (2) recommends everyone receive the most up to date COVID-19 vaccine if eligible. This includes individuals who have already contracted COVID-19 as the vaccines can provide additional protection and decrease the likelihood of hospitalization with a new infection (4). The goal of COVID-19 booster vaccination is to increase the immune response to the most recent COVID-19 variant to help limit the chance of severe illness (6).

The COVID-19 vaccines and boosters are important for college students to receive as they can minimize the effects of COVID-19 infection across campuses and communities. As of September 14, 2022, over 1,000 college and university campuses in the United States required COVID-19 vaccination, and nearly 300 required boosters, spanning 33 states and the District of Columbia (7). Given the decline in hospitalizations and deaths linked to COVID-19 and the availability of more effective tools to prevent and address COVID-19, the CDC has updated and simplified its recommendations for respiratory viruses, including influenza, RSV and COVID-19. As such, many college campuses have relaxed their COVID-19-related recommendations. Leidman et al. (8) found that 57.4% of all infections of those ranging in age from 0 to 24 years occurred in those aged 18–24 years. This suggests that young adults aged 18–24 may contribute considerably to the transmission of COVID-19, especially in comparison to those who are younger (8). As of October 16, 2023, the 18–29-year-old age group, which includes traditional college-age students, accounts for the largest number of cumulative COVID-19 cases compared to all other age groups (9).

College students are an important population to study regarding COVID-19 due to the increased chance of spreading the virus to other students, faculty, and staff on campus and within the surrounding community (10). Shared living spaces, dining halls, study rooms, campus activities, classrooms, etc. are spaces where COVID-19 can be transmitted easily due to close quarters in an indoor environment. These living conditions facilitate the spread of COVID-19 across campuses and throughout communities. Lu et al. (11) examined 30 U.S. campuses and their surrounding communities to see if there was a relationship between outbreaks on campus and then throughout the surrounding community. The rapid transmission of these localized campus outbreaks extended to the entire county, resulting in a surge of new infections within nearby communities in the initial weeks following the return of students to campus (11). Considering the benefits of the COVID-19 booster vaccines for reducing the likelihood of severe outcomes and post-COVID complications of COVID-19, it is important to examine factors that may influence college students’ decisions to receive the COVID-19 booster vaccine.

The health belief model (HBM) is an intrapersonal level theory that was developed to further understand an individual’s estimate of personal susceptibility to, and severity of, an illness, and the likelihood of being able to reduce the threat through personal action (12). The HBM consists of six primary constructs: perceived susceptibility, perceived severity, perceived benefits, perceived barriers, cues to action, and self-efficacy (13). In our study, perceived susceptibility was operationalized as an individual’s perception of the risk associated with getting COVID-19. Four items were used to assess this construct with a possible construct score range of 4–20. Higher scores suggest higher perceived susceptibility to getting COVID-19. Perceived severity was operationalized as a person’s perception about the seriousness of contracting COVID-19. Three items were included to assess perceived severity, with a possible construct score range of 3–15. Higher scores indicate higher perceived severity for COVID-19. Two of the perceived severity items were reverse coded. Perceived benefits was operationalized as a person’s perceptions of the positive consequences of receiving the COVID-19 booster vaccine in the next 6 months. The perceived benefits construct was assessed using four items with a possible score range of 4–20. Higher scores indicate more positive perceived consequences of receiving the COVID-19 booster vaccine. Perceived barriers was operationalized as an individual’s perceptions on the obstacles to getting the COVID-19 booster vaccine in the next 6 months. Perceived barriers was measured through five items, with a possible score range of 5–25. Higher scores indicate more perceived barriers to getting the COVID-19 vaccine. Cues to action was operationalized as stimuli that would trigger the person to get the COVID-19 booster vaccine. Cues to action was measured through five items with a possible score range of 5–25. Higher scores indicate more stimuli that would trigger the person to receive the COVID-19 booster. Self-efficacy was operationalized as an individual’s perceived confidence level that they could successfully get the COVID-19 booster in the next 6 months. Self-efficacy was measured using four items with a possible score range of 4–20. Higher scores indicate greater self-efficacy to get the COVID-19 vaccine in the next 6 months.

Although not a primary construct of the HBM, behavioral intention is commonly applied as a predictive antecedent construct to behavioral performance in HBM studies (14, 15). Together, these constructs are said to simultaneously influence an individual’s decision to engage in or refrain from a given behavior. The HBM may be an effective theoretical framework for examining the COVID-19 booster intentions and uptake among college students since it has successfully been applied to other vaccines such as the human papillomavirus in female university students, the flu vaccine, and the initial COVID-19 vaccine (16–22). For the purposes of this study, behavioral intention was operationalized as a person’s readiness to get the COVID-19 booster in the next 6 months. Behavioral intention was assessed through three items with a possible score range of 3–15.

The primary purpose of this study was to apply the health belief model (HBM) constructs in predicting COVID-19 booster behavioral intentions of college students. We also assessed COVID-19 vaccination status and the proportion of self-reported current or prior COVID-19 infections in the sample. The results of this study may help to determine if HBM is a viable framework for booster-related interventions with college students and to inform effective, theory-based health promotion programs to increase COVID-19 booster intentions and uptake among college students.

### Research questions

The following questions were investigated in this study:

1. What are the current COVID-19 vaccination rates among college students in the sample? This includes assessing the proportion of students who are (a) unvaccinated, (b) partially vaccinated, (c) fully vaccinated, and (d) boosted.
2. What proportion of the sample has self-reported currently or previously having COVID-19?

H1: The constructs of perceived susceptibility (β_1_), perceived severity (β_2_), perceived barriers (β_3_), cues to action (β_4_), perceived benefits (β_5_) self-efficacy (β_6_) combined will significantly predict behavioral intention to receive the COVID-19 booster in the next 6 months *(Y)* among college students.

## Materials and methods

This study utilized a survey which was designed to assess HBM-based predictors of COVID-19 booster vaccination intentions and aid in designing relevant COVID-19 booster vaccination interventions for college students. A non-experimental, cross-sectional study design was applied in this study which was approved by the university’s Institutional Review Board (IRB #22–0099).

### Population

The sample was comprised of college students attending a large public university located in the Southeastern region of the United States. The inclusion criteria were as follows: (a) part-time or full-time college students, (b) who currently reside in the United States, (c) are 18 years old and older, and (d) do not have a medical condition which prevents them from receiving vaccines. Only students who reported being vaccinated with the COVID-19 vaccine but who had not yet received the COVID-19 booster were asked to complete the HBM questions, as they were the only participants eligible for the booster vaccine. It is important to note that COVID-19 vaccination is not required at the institution where the data was collected.

### Instrumentation methodology

Instrument items were selected (23), modified (24), and/or developed by the research team based on extensive research on COVID-19 vaccination, COVID-19 booster vaccination, and health belief model-based literature (8, 11, 14, 25–27). We adapted to Shmueli’s (24) perceived susceptibility, perceived severity, perceived barriers, perceived benefits, and cues to actions scales that were previously validated. We also adapted Catalano et al.’s (23) previously validated scales for behavioral intentions and self-efficacy. The self-efficacy scale was adapted from the perceived behavioral control scale used in the Catalano et al. (23) study as these are very similar constructs. A few HBM items were slightly modified for reasons such as specifying the COVID-19 booster vaccine, using accurate terminology, applying a uniform 5-point Likert scale to each HBM scale, and using the correct timeframe (i.e., 6 months) for our study. For example, one cues to action item in the study was modified from, “The chances of me getting vaccinated against COVID-19 will increase if my GP recommends me” (24) to “The chances of me getting the COVID-19 booster vaccine will increase if primary care providers (e.g., doctor, nurse practitioner, physician assistant) recommend the booster vaccine.” Lastly, this item was modified from a 1–6 agreement scale to a 5-point Likert scale allowing for continuity across all HBM items and user ease. Another example of an item we modified was, “If I wanted to, I could get a COVID-19 booster vaccine in the next 12 months, even if my schedule is busy” (24). The timeframe was changed from 12 months to 6 months and the 1–6 agreement scale was changed to the 5-point Likert scale. One of the perceived susceptibility items was “I believe that if I do not get the COVID-19 booster vaccine, the likelihood of me getting infected with COVID-19 will increase.” Two of the perceived severity items were reverse coded, including “If I get infected with COVID-19, I do not think it will cause me significant suffering or complications” and “I believe that if I got infected with COVID-19, it would cause severe health problems.” One example of a perceived barriers item was “Getting a COVID-19 booster vaccine would be inconvenient.” One example of a behavioral intention item was “I will get the COVID-19 booster vaccine in the next 6 months.” Due to the timing of this study, a booster was defined as follows. “A COVID-19 booster vaccine is NOT the same as getting a 1-dose vaccine (ex. Johnson & Johnson or AstraZeneca) or a 2-dose vaccine (ex. Pfizer-BioNTech or Moderna). A booster shot is an additional dose of a vaccine to “boost” your immunity. A booster shot is taken AFTER completing the vaccine series.”

### Instrument components

There were four eligibility questions in the survey instrument. After completing these questions, eligible respondents were asked to complete between 13 and 42 questions, depending on how they answered the question about whether they had received any COVID-19 vaccine doses. The survey was designed to measure HBM constructs of perceived severity, perceived susceptibility, perceived benefits, perceived barriers, cues to action, self-efficacy, behavioral intention, as well as descriptive and demographic variables. A 5-point Likert scale was used in previous HBM studies [See (28, 29)] and applied in the current survey. A minimum of three items were developed for each HBM construct. The mean item score found for each HBM construct was used to provide an overall construct score.

### Data collection

Participants at a large public university located in the Southeastern region of the United States were recruited from six undergraduate programs/majors, four minors, a wellness course that all undergraduate students are required to take, and four graduate and/or graduate certificate programs, all of which are housed in the same academic unit. The first two authors contacted course instructors via e-mail and to provide an overview of the research study, procedures, and time requirements. The instructor was then asked to forward the recruitment email to students in their respective class (es). Time to complete the online Qualtrics™ instrument was estimated between 10 and 12 min, and participants were not compensated.

Students who were interested in the study clicked on the survey link embedded in the initial recruitment email, which directed them to the informed consent page via Qualtrics™. After reading the informed consent page, participants provided informed consent by selecting that they agreed to participate in the study. Respondents who gave consent answered screening/eligibility questions. Respondents who did not give consent to participate in the study were directed to the end of the survey. Respondents who qualified for the study were taken to the first page of the survey and they proceeded through the questions until they had completed the online questionnaire. The online survey was comprised of 28 HBM-based items (including behavioral intention), and eighteen closed-response items with some options for text entry which were used to obtain demographic and descriptive information.

### Data analyses

Data was analyzed using International Business Machines (IBM®) Statistical Package for Social Sciences (SPSS), Statistics version 25.0. Descriptive statistics were used to determine the characteristics of the sample and to answer research questions 1 and 2. Cronbach’s alpha measured internal consistency of each HBM construct, which determined how closely items relate to other items on the same scale (30). In this case, items are combined into a single scale and operationalized as a construct. As a pre-requisite step to address hypothesis 1, bivariate correlation coefficients were used to determine relationships between the HBM constructs and behavioral intention to get the COVID-19 booster vaccine in the next 6 months. Furthermore, stepwise multiple regression was applied to build the model and assess its predictive validity for research question 3. The independent variables were the HBM constructs, and the dependent variable was behavioral intention. Stepwise method criteria required the probability of the *F*-statistic to be less than or equal to 0.05 to enter a predictor in the model and greater than or equal to 0.10 to remove a predictor from the model.

## Results

The survey was open for responses for the first 3 weeks of March 2022. A total of 290 people clicked on the survey link. Of those, 289 respondents gave their consent to participate in the study. One respondent did not consent to participate and was excluded from the study. Four additional cases were excluded from analysis because they failed to answer more than 20% of the questions. The final sample consisted of 285 respondents for a survey completion rate of 98.3%.

### Demographic characteristics of the sample

The majority of the sample were females (84.6%; *n* = 241) between 18 and 24 years of age (89.8%; *n* = 256). Approximately 65% (*n* = 186) of the sample reported living off-campus or in other non-university housing. Additionally, a majority of the sample was comprised of undergraduate students (94%; *n* = 268). A total of 80.4% (*n* = 229) of the sample had received the COVID-19 vaccine and 51.2% (*n* = 146) of respondents reported having received the Pfizer-BioNTech vaccine specifically. Nearly half of the sample (45.3%; *n* = 129) reported having received two COVID-19 vaccines. A summary of the demographic frequency statistics for the sample is included in Table 1.

**Table 1:** Summary of demographic frequency statistics for the primary sample (*N* = 285).

Variable	*n*	%
Gender identity		
Male	40	14.0
Female	241	84.6
Trans-male	1	0.4
Gender variant/non-conforming	2	0.7
Prefer not to say	1	0.4
Age (in years)		
18–24	256	89.8
25–34	20	7.0
35–44	5	1.8
45–54	3	1.1
55–64	1	0.3
Academic standing		
First year undergraduate	41	14.1
Second year undergraduate	63	22.1
Third year undergraduate	104	36.5
Fourth year undergraduate	50	17.5
Fifth year undergraduate	10	3.5
Graduate	16	5.6
Non-degree seeking	1	0.4
Ethnicity		
Not of Hispanic, Latinx, or Spanish origin	259	90.9
Mexican, Mexican American, or Chicano	9	3.2
Puerto Rican	3	1.1
Cuban	2	0.7
Another	12	4.2
Race		
American Indian or Alaska Native	8	2.8
Asian Indian	5	1.8
Black or African American	9	3.2
Chinese	2	0.7
Filipino	2	0.7
Japanese	1	0.4
Korean	1	0.4
Other Asian	2	0.7
White	258	90.5
Other	4	1.4
Prefer not to answer	5	1.8
Living		
Campus or university housing	82	28.8
Parent/guardian/other family member’s home	17	6.0
Off-campus or other non-university housing	186	65.3

### Theoretical construct analyses

A summary of the mean, standard deviation, possible range, observed range, and Cronbach’s *a* for each of the HBM-based subscales is included in Table 2. All the subscales demonstrated satisfactory internal consistency.

**Table 2:** Ranges, means, standard deviations, and Cronbach’s alpha coefficients for the health belief model constructs (*N* = 114)*.

	Descriptive statistics				Reliability statistics
Construct	Possible range	Observed range	*M*	SD	Cronbach’s *α*
Behavioral intention	3–15	3–15	9.66	4.29	0.978
Perceived susceptibility	4–20	4–20	11.24	4.13	0.914
Perceived severity	3–15	3–13	6.27	2.58	0.779
Perceived benefits	4–20	4–20	12.59	4.07	0.839
Perceived barriers	5–25	5–20	11.58	3.48	0.616
Cues to action	5–25	5–25	16.92	4.44	0.819
Self-efficacy	4–20	9–20	17.48	2.74	0.871

^*^The sample consists of those who were deemed “fully vaccinated” but who had not yet received the booster vaccine.

### Research question 1

For research question 1, over three quarters (81.4%, *n* = 232) of the sample reported being fully vaccinated, 2.1% (*n* = 6) reported being partially vaccinated, and 16.5% (*n* = 47) reported having not received any doses of the vaccine. Furthermore, 53.4% (*n* = 124) of students who self-reported being fully vaccinated also reported receiving the booster vaccine.

### Research question 2

For research question 2, nearly half (49.1%, *n* = 140) of the sample self-reported currently or previously having COVID-19 as confirmed by a positive test result. Furthermore, 9.8% of the sample (*n* = 28) self-reported maybe having COVID-19, meaning they had symptoms consistent with COVID-19, but it was not confirmed by a test.

### Bivariate relationships between HBM constructs and behavioral intention

Perceived susceptibility, perceived barriers, perceived benefits, cues to action, and self-efficacy all had significant correlations with behavioral intention to receive the COVID-19 booster in the next 6 months. Perceived severity did not have a statistically significant relationship with behavioral intention. A summary of the Pearson product–moment correlations for the independent and dependent variables are presented in Table 3.

**Table 3:** Pearson product–moment correlation coefficients for the health belief model constructs (*N* = 114).

	Constructs	7	6	5	4	3	2	1
1.	Behavioral intention	0.239*	0.667**	−0.247**	0.777**	0.158	0.582**	–
2.	Perceived susceptibility	0.016	0.556**	−0.133	0.649**	0.223*	–	
3.	Perceived severity	−0.221*	0.176	−0.034	0.229*	–		
4.	Perceived benefits	0.279**	0.658**	−0.343**	–			
5.	Perceived barriers	−0.400**	−0.146	–				
6.	Cues to action	0.112	–					
7.	Self-efficacy	–						

*p < 0.01; **p < 0.001.

### Hypothesis 1 – HBM-based predictors of behavioral intention

Lastly, we tested the significance of perceived susceptibility, perceived severity, perceived barriers, perceived benefits, cues to action, and self-efficacy to predict behavioral intention to receive the COVID-19 booster vaccine in the next 6 months among college students. Multiple linear regression using the stepwise method modeled the HBM constructs on behavioral intention. The probability of *F* statistic to enter a predictor in the model was set *a priori* to less than or equal to 0.05 and removal of a predictor as greater than or equal to 0.10. Results of the stepwise multiple regression indicated the HBM constructs of perceived benefits (*β* =0.596; *p* < 0.001) and cues to action (*β* =0.275; *p* < 0.001) were significant predictors of respondents’ behavioral intention to receive the COVID-19 booster vaccine in the next 6 months. When combined, the significant predictors at step 2 accounted for 64.6% [*R*^2^ = 0.646, *F* (2, 111 = 101.331, *p* < 0.001)] of the variance in behavioral intention to get the COVID-19 booster vaccine in the next 6 months. The final prediction equation suggested respondents’ behavioral intention to get the COVID-19 booster vaccine in the next 6 months = −2.759 + 0.596 (perceived benefits) + 0.275 (cues to action). Consequently, null hypothesis 1 was rejected. Table 4 provides a summary of the parameter estimates for the model with all operationalized HBM constructs.

**Table 4:** Stepwise regression model predicting COVID-19 Booster vaccination intentions of partially or fully vaccinated college students (*N* = 114).

Variable	*B*	SE B	*β*	*T*	*p*	*R*^2^ Change
Step 1: included variables						
Constant	−0.671	0.831		−0.807	0.099	
Perceived benefits	0.820	0.063	0.777	13.048	0.000	0.603
Step 1: excluded variables						
Perceived susceptibility	0.135			1.746	0.084	
Perceived severity	−0.021			−0.344	0.732	
Perceived barriers	0.022			0.351	0.726	
Self-efficacy	0.024			0.391	0.696	
Cues to action	0.275			3.670	0.000	
Step 2: included variables						
Constant	−2.759	0.973		−2.837	0.005	
Perceived benefits	0.629	0.079	0.596	7.943	0.000	0.603
Cues to action	0.266	0.072	0.275	3.670	0.000	0.043
Step 2: excluded variables						
Perceived susceptibility	0.078			1.021	0.310	
Perceived severity	−0.029			−0.490	0.625	
Perceived barriers	−0.003			−0.044	0.965	
Self-efficacy	0.046			0.779	0.438	

Step 2 total *R*^2^ = 0.646.

## Discussion

The COVID-19 vaccination rates among the students in this sample are similar to national estimates, with 81.4% of the survey respondents being fully vaccinated, 2.1% partially vaccinated, and 16.5% unvaccinated. In comparison, 85.2% of college students in a nationally representative survey indicated that they had been vaccinated against COVID-19 (31). According to the CDC (26), 79.1% of those over the age of 18 in the United States are currently fully vaccinated and 20.5% of the population have also received an updated bivalent booster. In our study, 16.5% (*n* = 47) of students self-reported being unvaccinated. Although the majority of college students are fully vaccinated, there is a substantial segment of the student population that remains at higher risk for COVID-19 infections and more serious complications because they are unvaccinated. Furthermore, among those who self-reported being unvaccinated in our sample, 87.2% (*n* = 41) indicated that they did not plan on getting the COVID-19 vaccine in the next 6 months. In contrast, slightly over half (53.4%) of students who self-reported being fully vaccinated reported receiving the booster vaccine. This indicates that nearly half of the fully vaccinated sub-sample had not been boosted, which places them at higher risk for serious complications from COVID-19 infection. Receiving an updated COVID-19 vaccine helps prime the immune system and is necessary to protect against serious illness (31). Furthermore, an updated booster can help the immune system recognize new variants of the virus arising from mutation. The large proportion of non-boosted individuals in our study point to a need to further encourage college students to become fully vaccinated and boosted.

Almost half (49.1%) of the sample reported currently or previously having COVID-19 as confirmed by a positive test result. Additionally, 9.8% of the sample selected “maybe (e.g., I have had symptoms consistent with COVID-19, but it was not confirmed by a test).” The COVID-19 infection percentages in this university sample were higher than previous national data which reported 43.3% of the country had been infected by the virus as of January 2022 (33). The findings for this study, while interesting, are not particularly surprising since the data for the present study was collected a few months later, allowing additional time for the Omicron wave to peak in the location where the data was collected. In Fall 2021, the National College Health Assessment II (NCHA II) survey (33) revealed that 19.0% of college students self-reported having tested positive for COVID-19 compared to 45% in Fall 2022 (31). The NCHA II data that was collected in Fall 2021 likely did not account for the Omicron wave that began to sweep through the US in early December 2021. This may explain the larger percentage of self-reported COVID-19 infections in the Fall 2022 NCHA II survey results and our study.

Although perceived susceptibility was not a statistically significant predictor of behavioral intention in the multiple linear regression model, there is room for improvement in this domain among college students. The mean value of this construct fell slightly above mid-range (*M* = 11.24 units out of 20). Interventions should attempt to change individuals’ perceptions of risk associated with acquiring COVID-19. To increase perceived susceptibility among college students, it is important to focus on specifics such as the likelihood of getting infected with COVID-19 without an updated COVID-19 vaccine, and how easily transmissible it is to others, (25, 34). Young adults ages 18–24 years may contribute more to the spread of COVID-19 in comparison to the other age groups, which highlights the important role that traditional college-aged students play in COVID-19 transmission within local communities and beyond (8). An analysis of European data calculated a COVID-19 reproduction number (R_0_) of 2.2 (95% CI: 1.9–2.6), which estimates the average number of new cases resulting from a single infected individual in a non-immune population (34). An R_0_ value greater than 1 implies exponential spread of the disease, which further illustrates the contagiousness of the disease (35). Based on this data, an estimated 55% of a European population would need to develop immunity against COVID-19 to curtail an outbreak. Studies from the CDC state that unvaccinated people had 13.9 times the risk for infection compared with fully vaccinated people who received booster doses (34). Unvaccinated people had 4.0 times the risk for infection compared with fully vaccinated people without booster doses (33). This information could be presented to college students as part of future educational interventions intended to increase perceived susceptibility to getting COVID-19.

Perceived severity was also not a statistically significant predictor of behavioral intention in the multiple linear regression model. The perceived severity construct had a mean score that fell below mid-range (*M* = 6.27 units out of 15), which indicates the need to increase college students’ perceived severity of the risks and/or complications associated with getting COVID-19. Unvaccinated people had 53.2 times the risk for COVID-19 associated death compared with fully vaccinated people who received booster doses (33). Unvaccinated people had 12.7 times the risk for COVID-19 associated death compared with fully vaccinated people without booster doses (33). A more recent study found that the bivalent booster offers enhanced protection against COVID-19-associated mortality (36). One strategy to increase the mean of this construct would be to inform students on the severe consequences associated with COVID-19, and the impacts it can have on them personally. It would be important to educate students on the hospitalization and death rates of their age group to convey the severity of COVID-19, particularly to those not up to date with their vaccinations, including young adults. Another strategy could be to focus on non-medical consequences of contracting COVID-19. According to Washburn (37), perceived severity can also be based on personal beliefs about how the health condition might impact an individual’s life. Non-medical consequences applicable to college students could be related to academic achievement, employment, and sense of belonging. For instance, in Fall 2022, the NCHA II survey revealed that 1.2% of students reported that having COVID-19 in the past 12 months negatively impacted their performance in a class, and 1.5% said it delayed progress toward their degree (31). Students may also be motivated to get the COVID-19 booster to avoid getting sick and to prevent disruptions to school, work, family responsibilities, and social activities.

The perceived barriers construct was not a statistically significant predictor of behavioral intention within the multiple linear regression model. The mean value was 11.58 units out of a possible 25, with higher values representing more perceived barriers to getting the COVID-19 booster vaccine. This finding indicates that there is still room to reduce perceived barriers to getting the COVID-19 vaccine within the college student population. To reduce barriers, interventions could be tailored to address how to overcome specific barriers (i.e., lack of transportation, difficulty finding an appointment, fear, etc.) to receiving the COVID-19 booster vaccine (38). One strategy to overcome some common barriers is for universities to offer free booster vaccination clinics on campus (including satellite and branch campuses), which would be a convenient, accessible, and affordable option for students.

Self-efficacy was not a statistically significant predictor of behavioral intention in the multiple linear regression model. However, the mean score value for self-efficacy was high, 17.48 units out of a possible 20. This is consistent with findings from another study reporting that participants were confident in their ability to get COVID-19 vaccines, even though self-efficacy and vaccination intention were not statistically significant (26). Interventions should continue emphasizing college students’ self-ability and confidence to receive the booster, despite minor obstacles. Strategies to foster self-efficacy include having college students set goals they want to achieve and adjusting them until they are fully confident in their ability to get the booster vaccine. One study found that feeling support from peers, family, and doctors increased HPV vaccine self-efficacy for undergraduate students (39). Feeling supported by these groups may also translate to receiving the COVID-19 booster.

Constructs that were found to have statistically significant relationships with behavioral intention using the stepwise linear regression model were perceived benefits and cues to action. This finding is similar to another study where perceived benefits and cues to action were also found to be significant predictors of intention to get vaccinated against COVID-19, with the addition of perceived severity (24). The perceived benefits construct was the strongest predictor of behavioral intention to receive the COVID-19 booster vaccine. Perceived benefits can be increased by continuing to promote benefits associated with receiving the booster vaccine. Underscoring the percentages of those who have less severe symptoms of COVID-19 after getting boosted along with fewer COVID-19 associated emergency department encounters are both important benefits resulting from getting the COVID-19 booster vaccine. Thompson et al. (40) found that the booster was highly effective at preventing these emergency department encounters, decreasing them by 90% during the Delta and Omicron-predominant periods. A CDC study published in 2023 found that unvaccinated people were 14.1 times more likely to die from COVID-19 compared to recipients of the bivalent vaccine (36). Furthermore, Johnson et al. (36) found that unvaccinated people were 2.8 times more likely to get infected with COVID-19, compared to those who had received bivalent doses. This demonstrates that individuals who are vaccinated and/or boosted have greater protection against infection and mortality in comparison to unvaccinated people, an important factor to stress during future interventions. Another important benefit to emphasize is protecting others from getting sick. A study conducted in Europe found that college students are largely concerned about others contracting COVID-19 and that this perception is associated with adherence to safety measures, including vaccination (41). This study also found that cues to action was a statistically significant predictor of behavioral intention to receive the COVID-19 booster vaccine (41). Another study in Israel recommended having the Ministry of Health and general practitioners recommend COVID-19 vaccination (24). This may also translate to college student populations. For example, it may be helpful for well-respected healthcare providers (e.g., medical doctors, nurse practitioners, physician assistants) to hold educational sessions promoting the importance of receiving the COVID-19 booster vaccine.

Sharing booster recommendations from governmental public health agencies, such as the CDC, may serve as cues to action for college students. Previously, there was the belief that having universities require the COVID-19 booster vaccine would serve as a major cue to action for students to getting boosted, otherwise they would not be allowed back on campus. Many colleges and universities implemented this action (7), but now these types of mandates are no longer supported (42). The COVID-19 vaccines have not yet risen to the level of acceptance as other required immunizations for attending students. For example, in the state of North Carolina, college students are required by law to receive diphtheria, tetanus, and pertussis, polio, measles, mumps, rubella, hepatitis B, and varicella immunizations (43).

Other strategies that showed promise for reducing vaccine hesitancy or increasing COVID-19 vaccine uptake included emphasizing personal benefit in communications and delivering text reminders for vaccination appointments (44). For example, universities can implement an Immunization Information System (IIS) as recommended by the CDC to increase adult immunization rates (45). An IIS could provide text reminders to students about personal benefits of vaccination and scheduling vaccination appointments. A similar IIS designed and tested in Indonesia resulted in increased vaccination rates (46). Further, this IIS incorporated relevant articles, which are also known to serve as external cues to action under the assumptions of the HBM.

College students in this sample had moderate behavioral intentions to get the COVID-19 booster vaccine in the next 6 months, with a mean value of 9.66 units out of a possible maximum score of 15 units. These findings are similar to another study which reported COVID-19 booster vaccination intentions to be high (47). For participants who report some amount of behavioral intention, interventionists may promote COVID-19 booster vaccine series completion by asking participants to indicate when, where, and how they plan to get each dose of the COVID-19 booster vaccine within the next 6 months (48). This technique is referred to as an implementation intention, which has demonstrated effectiveness with increasing individuals’ ability to initiate and maintain health behaviors (49). Implementation intention is one of the most effective strategies available to facilitate the transition from intention to behavior (50). This technique may support participants who have some level of behavioral intention to follow through to get the COVID-19 booster within the next 6 months.

Overall, these results illustrate that some constructs of the HBM, namely perceived benefits and cues to action, are useful for predicting COVID-19 booster intentions among college students. Although the other HBM constructs were not statistically significant, the full HBM framework may still be viable as there is room to increase college students’ perceived susceptibility and perceived severity to COVID-19 and decrease perceived barriers to getting the COVID-19 vaccine. The only exception was self-efficacy, which had a high mean score, indicating that overall, college students’ felt confident in their ability to get a COVID-19 booster vaccine. This is not particularly surprising since this sub-sample had already been successful with receiving one or more doses of the COVID-19 vaccine. Therefore, the findings indicate that practitioners should aim to maintain or further increase college students’ self-efficacy.

Overall, the HBM constructs are relevant to college students’ decision-making processes regarding COVID-19 booster intentions and may be used to inform interventions on university campuses. A mixed methods study done in Europe found that university students weighed costs and benefits when making decisions and forming attitudes about COVID-19 safety measures including vaccination behavior which aligned well with the HBM framework (41).

### Limitations and future research recommendations

As with any study, this investigation has some limitations that must be taken into consideration when interpreting the results. First, this was a non-experimental, cross-sectional study design; therefore, there is an antecedent-consequent bias, and it is difficult to derive causal relationships from analysis. Second, only intrapersonal-level factors were investigated. Social, economic, environmental, or political factors that may influence behavioral intention to get the COVID-19 booster vaccine were not examined. Prospective research is needed to better understand the temporal relationships between constructs. These studies could help to illuminate the relationship between behavioral intention to get the COVID-19 booster vaccine and receiving the COVID-19 booster vaccination among college students. Thirdly, the survey was conducted asynchronously online, which does not allow for the subject to ask questions. To prevent skewed responses from this method, the recruitment email(s) included a statement that participants were able to email the PI or research team at any time with questions they had about the survey. The study was conducted on one university campus and distributed only to those majoring or taking classes housed in a large health and human sciences unit. The majority of students enrolled in classes housed in this unit are health-related; however, some classes within this unit are open to all students enrolled at the university. This may result in selection bias; therefore, the results cannot be extrapolated beyond the sample. To reduce selection bias in future studies, it would be beneficial to employ random sampling of university students across a wide array of disciplines. Additionally, to facilitate a larger, more representative sample, random sampling methods could be applied in future studies. For example, the survey could be distributed to a random sample of colleges and universities at the state, regional, or national level.

Another limitation is that we examined COVID-19 booster behavioral intentions rather than uptake as the dependent variable in the model. Although behavioral intention is a precursor to actual behavior, we did not follow up with participants after 6 months to determine if behavioral intention accurately predicted COVID-19 booster uptake. Future studies may examine this temporal relationship using path analysis. One important note is that the CDC is now using the term updated COVID-19 vaccine rather than booster, similar to the description used for the annual flu vaccine.

Lastly, future research is needed to identify the most effective COVID-19 vaccine messages and messengers, the most important settings and channels to reach the targeted audience, and the most effective strategies for the design and implementation of up-to-date COVID-19 vaccine educational interventions targeting college students.

## Conclusion

This study was one of the first investigation to examine HBM constructs in predicting COVID-19 booster intentions among college students in the United States. Overall, the findings illustrate that HBM is a viable framework for predicting COVID-19 booster intentions among college students. Perceived benefits and cues to action were statistically significant predictors of college students’ behavioral intention to get the COVID-19 booster vaccination within the next 6 months. Additionally, this study examined vaccination status and previous or current COVID-19 infection rates. Findings from this study also indicate that many college students are at risk for more severe complications of COVID-19 infections due to not being fully vaccinated or boosted. Practitioners developing HBM-based interventions to enhance COVID-19 booster intentions among college students should tailor health promotion strategies that target perceived benefits and cues to action. Although some of the HBM constructs were not statistically significant in the prediction model, they should not be entirely discounted in practice. Instead, health promotion practitioners should focus on supplemental strategies to improve those domains in college students.

## Data Availability

The data for this manuscript is not publicly available because the Institutional Review Board (IRB) stipulated that this data is accessible only to personnel listed in the protocol. For further inquiries, the data that support the findings of this study are available from the lead author upon reasonable request. (Hannah Priest Catalano, catalanoh@uncw.edu).

## References

[1] Centers for Disease Control and Prevention. End of the federal COVID-19 public health emergency (PHE) declaration (2023). Available at: https://www.cdc.gov/coronavirus/2019-ncov/your-health/end-of-phe.html. Accessed October 16, 2023.

[2] Centers for Disease Control and Prevention. Interim clinical considerations for use of COVID-19 vaccines in the United States (2023). Available at: https://www.cdc.gov/vaccines/covid-19/clinical-considerations/interim-considerations-us.html Accessed October 16, 2023.

[3] Centers for Disease Control and Prevention. How to protect yourself and others (2023). Available at: https://www.cdc.gov/coronavirus/2019-ncov/prevent-getting-sick/prevention.html. Accessed October 16, 2023.

[4] Centers for Disease Control and Prevention. Benefits of getting a COVID-19 vaccine (2023). Available at: https://www.cdc.gov/coronavirus/2019-ncov/vaccines/vaccine-benefits.html. Accessed October 16, 2023.

[5] Centers for Disease Control and Prevention. Overview of COVID-19 vaccines (2023). Available at: https://www.cdc.gov/coronavirus/2019-ncov/vaccines/different-vaccines/overview-COVID-19-vaccines.html. Accessed June 13, 2023.

[6] Rosenblum HG Wallace M Godfrey M Roper LE Hall E Fleming-Dutra KE. Interim recommendations from the advisory committee on immunization practices for the use of bivalent booster doses of COVID-19 vaccines — United States, October 2022. MMWR Morb Mortal Wkly Rep. (2022) 71:1436–41. doi: 10.15585/mmwr.mm7145a2, PMID: 36355612 PMC9707353

[7] Castillo E Appleby C. These are the colleges requiring vaccine boosters now (2023). Available at: https://www.bestcolleges.com/news/2021/12/14/what-colleges-require-covid-vaccine-booster-omicron/. Accessed June 21, 2023.

[8] Leidman E Duca LM Omura JD Proia K Stephens JW Sauber-Schatz EK. COVID-19 trends among persons aged 0–24 years — United States, march 1–December 12, 2020. MMWR Morb Mortal Wkly Rep. (2021) 70:88–94. doi: 10.15585/mmwr.mm7003e1, PMID: 33476314 PMC7821770

[9] Centers for Disease Control and Prevention. Demographic trends of COVID-19 cases and deaths in the US reported to CDC (2023). Available at: https://covid.cdc.gov/covid-data-tracker/#demographics Accessed October 16, 2023.

[10] Walke HT Honein MA Redfield RR. Preventing and responding to COVID-19 on college campuses. JAMA. (2020) 324:1727–8. doi: 10.1001/jama.2020.20027, PMID: 32991681 PMC9648565

[11] Lu H Weintz C Pace J Indana D Linka K Kuhl E. Are college campuses superspreaders? A data-driven modeling study. Comput Methods Biomech Biomed Engin. (2021) 24:1136–45. doi: 10.1080/10255842.2020.1869221, PMID: 33439055

[12] Llewellyn CD Ayers S McManus C Newman S Petrie KJ Revenson TA. Cambridge handbook of psychology, health and medicine. 3rd ed. Cambridge, England: Cambridge University Press (2019).

[13] Rosenstock IM. Historical origins of the health belief model. Health Ed Monographs. (1974) 2:328–35. doi: 10.1177/109019817400200403

[14] Tsai F-J Hu Y-J Chen C-Y Tseng C-C Yeh G-L Cheng J-F. Using the health belief model to explore nursing students’ relationships between COVID-19 knowledge, health beliefs, cues to action, self-efficacy, and behavioral intention: a cross-sectional survey study. Medicine. (2021) 100:e25210. doi: 10.1097/md.0000000000025210 33726016 PMC7982209

[15] Zampetakis LA Melas C. The health belief model predicts vaccination intentions against COVID-19: a survey experiment approach. Appl Psychol Health Well Being. (2021) 13:469–84. doi: 10.1111/aphw.12262, PMID: 33634930 PMC8014148

[16] Donadiki EM Jiménez-García R Hernández-Barrera V Sourtzi P Carrasco-Garrido P López de Andrés A. Health belief model applied to non-compliance with HPV vaccine among female university students. Public Health. (2014) 128:268–73. doi: 10.1016/j.puhe.2013.12.004, PMID: 24529635

[17] Mahmud I Kabir R Rahman MA Alradie-Mohamed A Vinnakota D Al-Mohaimeed A. The health belief model predicts intention to receive the COVID-19 vaccine in Saudi Arabia: results from a cross-sectional survey. Vaccine. (2021) 9:864. doi: 10.3390/vaccines9080864, PMID: 34451991 PMC8402432

[18] Mercadante AR Law AV. Will they, or Won’t they? Examining patients’ vaccine intention for flu and COVID-19 using the health belief model. Res Social Adm Pharm. (2021) 17:1596–605. doi: 10.1016/j.sapharm.2020.12.012, PMID: 33431259 PMC7833824

[19] Romano JL. Politics of prevention: reflections from the COVID-19 pandemic. J Prev Health Promot. (2020) 1:34–57. doi: 10.1177/2632077020938360, PMID: 38603060 PMC7358972

[20] Wong LP Alias H Wong P-F Lee HY Abu BS. The use of the health belief model to assess predictors of intent to receive the COVID-19 vaccine and willingness to pay. Hum Vaccin Immunother. (2020) 16:2204–14. doi: 10.1080/21645515.2020.1790279, PMID: 32730103 PMC7553708

[21] Wong MCS Wong ELY Huang J Cheung AWL Law K Chong MKC. Acceptance of the COVID-19 vaccine based on the health belief model: a population-based survey in Hong Kong. Vaccine. (2021) 39:1148–56. doi: 10.1016/j.vaccine.2020.12.083, PMID: 33461834 PMC7832076

[22] Limbu YB Gautam RK Pham L. The health belief model applied to COVID-19 vaccine hesitancy: a systematic review. Vaccine. (2022) 10:1–13. doi: 10.3390/vaccines1006973 PMC9227551 35746581

[23] Catalano HP Richards K Shaw KHH Catalano M. Applying the theory of planned behavior to predict COVID-19 booster vaccination intentions of college students. J Am Coll Heal. (2023):1–10. doi: 10.1080/07448481.2023.2228425, PMID: 37437193

[24] Shmueli L. Predicting intention to receive COVID-19 vaccine among the general population using the health belief model and the theory of planned behavior model. BMC Public Health. (2021) 21:804. doi: 10.1186/s12889-021-10816-7, PMID: 33902501 PMC8075011

[25] Alsulaiman SA Rentner TL. The use of the health belief model to assess U.S. college students’ perceptions of COVID-19 and adherence to preventive measures. J Public Health Res. (2021) 10:4. doi: 10.4081/jphr.2021.2273, PMID: 34340300 PMC8561461

[26] Chu H Liu S. Integrating health behavior theories to predict American’s intention to receive a COVID-19 vaccine. Patient Educ Couns. (2021) 104:1878–86. doi: 10.1016/j.pec.2021.02.031, PMID: 33632632 PMC7889032

[27] Centers for Disease Control and Prevention. COVID Data Tracker (2023). Available at: https://covid.cdc.gov/covid-data-tracker/#vaccinations_vacc-people-booster-percent-pop5. Accessed June 15, 2023.

[28] Diddana TZ Kelkay GN Dola AN Sadore AA. Effect of nutrition education based on health belief model on nutritional knowledge and dietary practice of pregnant women in Dessie town, Northeast Ethiopia: a cluster randomized control trial. J Nutr Metab. (2018) 2018:1–10. doi: 10.1155/2018/6731815, PMID: 30034866 PMC6033240

[29] Msengi IG. Development and evaluation of innovative recycling intervention program using the health belief model (HBM). Open J Prev Med. (2019) 9:29–41. doi: 10.4236/ojpm.2019.94004

[30] What does Cronbach’s alpha mean? Available at: https://stats.oarc.ucla.edu/spss/faq/what-does-cronbachs-alpha-mean/. Accessed June 21, 2023.

[31] American College Health Association. Reference group data report – Fall 2022. (2022). Available at: https://www.acha.org/documents/ncha/NCHA-III_FALL_2022_REFERENCE_GROUP_DATA_REPORT.pdf Accessed June 19, 2023

[32] Centers for Disease Control and Prevention. CDC Covid data tracker. (2022). Available at: https://covid.cdc.gov/covid-data-tracker/#national-lab Accessed June 26, 2023.

[33] American College Health Association. Reference group data report – Fall 2021. (2022). Available at: https://www.acha.org/documents/ncha/NCHA-III_FALL_2021_REFERENCE_GROUP_DATA_REPORT.pdf Accessed June 19, 2023

[34] Johnson AG Amin AB Ali AR Hoots B Cadwell BL Arora S. COVID-19 incidence and death rates among unvaccinated and fully vaccinated adults with and without booster doses during periods of delta and omicron (2022). Available at: https://www.cdc.gov/mmwr/volumes/71/wr/pdfs/mm7104e2-H.pdf Accessed June 26, 2023. 10.15585/mmwr.mm7104e2 PMC9351531 35085223

[35] Locatelli I Trächsel B Rousson V. Estimating the basic reproduction number for COVID-19 in Western Europe. PLoS One. (2021) 16:e0248731. doi: 10.1371/journal.pone.0248731, PMID: 33730041 PMC7968714

[36] Johnson AG Linde L Ali AR De Santis A Ms MS Adam C. COVID-19 incidence and mortality among unvaccinated and vaccinated persons aged ≥12 years by receipt of bivalent booster doses and time since (2022). Available at: https://www.cdc.gov/mmwr/volumes/72/wr/pdfs/mm7206a3-H.pdf 10.15585/mmwr.mm7206a3 PMC9925136 36757865

[37] Washburn L, Understanding the health belief model (2020). Available at: https://extension.tennessee.edu/publications/Documents/W931-C.pdf Accessed June 21, 2023.

[38] Burke PF Masters D Massey G. Enablers and barriers to COVID-19 vaccine uptake: an international study of perceptions and intentions. Vaccine. (2021) 39:5116–28. doi: 10.1016/j.vaccine.2021.07.056, PMID: 34340856 PMC8299222

[39] Stout ME Christy SM Winger JG Vadaparampil ST Mosher CE. Self-efficacy and HPV vaccine attitudes mediate the relationship between social norms and intentions to receive the HPV vaccine among college students. J Community Health. (2020) 45:1187–95. doi: 10.1007/s10900-020-00837-5, PMID: 32418009 PMC7606315

[40] Thompson MG Natarajan K Irving SA Rowley EA Griggs EP Gaglani M. Effectiveness of a third dose of mRNA vaccines against COVID-19 associated emergency department and urgent care encounters and hospitalizations among adults during periods of Delta and omicron variant predominance – VISION network, 10 states, august 21 – January 2022. Morb Mortal Wkly Rep. (2022) 71:139–45. doi: 10.15585/mmwr.mm7104e3, PMID: 35085224 PMC9351525

[41] Kulcar V Straganz C Kreh A Siller H File N Canazei M. University students’ adherence and vaccination attitudes during the COVID-19 pandemic: focusing on costs and benefits. Appl Psychol Health Well Being. (2022) 14:572–90. doi: 10.1111/aphw.12320, PMID: 34734472 PMC9297983

[42] Eyal N Wynia MK Harter TD DeBruin D Eberl JT. Why dropping most COVID-19 vaccine mandates is now ethical. Health Affairs Forefront. (2023) 14:572–90. doi: 10.1377/forefront.20231206.51079

[43] Branch NCI. NC DPH, WCH, Immunization: Schools and childcare facilities. Available at: https://immunization.dph.ncdhhs.gov/schools/collegesuniversities.htm Accessed June 21, 2023.

[44] Batteux E Mills F Jones LF Symons C Weston D. The effectiveness of interventions for increasing COVID-19 vaccine uptake: a systematic review. Vaccine. (2022) 10:3. doi: 10.3390/vaccines10030386, PMID: 35335020 PMC8949230

[45] Centers for Disease Control and Prevention. About ISS (2019). Available at: https://www.cdc.gov/vaccines/programs/iis/index.html Accessed June 23, 2023.

[46] Rahmadhan MAWP Azizi MI Handayani PW Monicha A. Design of a reminder and recall system in a contact tracing application to support coronavirus booster vaccination. Healthc Inform Res. (2023) 29:93–102. doi: 10.4258/hir.2023.29.2.93, PMID: 37190733 PMC10209730

[47] Hagger MS Hamilton K. Predicting COVID-19 booster vaccine intentions. Appl Psychol Health Well Being. (2022) 14:819–41. doi: 10.1111/aphw.12349, PMID: 35193171 PMC9111247

[48] Sheeran P Orbell S. Using implementation intentions to increase attendance for cervical cancer screening. Health Psychol. (2000) 19:283–9. doi: 10.1037/0278-6133.19.3.283, PMID: 10868773

[49] Gollwitzer PM. Implementation intentions: strong effects of simple plans. Am Psychol. (1999) 54:493–503. doi: 10.1037/0003-066x.54.7.493

[50] Centers for Disease Control and Prevention. (2023). Stay up to date. Available at: https://www.cdc.gov/coronavirus/2019-ncov/vaccines/stay-up-to-date.html Accessed October 16, 2023.

